# Case report: Varicella zoster virus encephalitis following COVID-19 vaccination in an immunocompetent individual

**DOI:** 10.1016/j.heliyon.2024.e28703

**Published:** 2024-03-30

**Authors:** Sanaz Rezaeian, Fatemeh Rahmanian, Zohre Rajabpour, Ali Taghipour, Mirza Ali Mofazzal Jahromi, Abdolvahab Rahmanian, Heshmatollah Shakeri, Navid Kalani, Maryam Jalali Jahromi, Amir Abdoli

**Affiliations:** a Student Research Committee, Jahrom University of Medical Sciences, Jahrom, Iran; b Zoonoses Research Center, Jahrom University of Medical Sciences, Jahrom, Iran; c Department of Internal Medicine, Jahrom University of Medical Sciences, Jahrom, Iran; d Department of Immunology, Jahrom University of Medical Sciences, Jahrom, Iran; e Department of Advanced Medical Sciences & Technologies, Jahrom University of Medical Sciences, Jahrom, Iran; f Department of Infectious Disease, Jahrom University of Medical Sciences, Jahrom, Iran; g Research Center for Social Determinants of Health, Jahrom University of Medical Sciences, Jahrom, Iran; h Department of Neurology, Jahrom University of Medical Sciences, Jahrom, Iran; i Department of Medical Parasitology and Mycology, Jahrom University of Medical Sciences, Jahrom, Iran

**Keywords:** VZV, COVID-19, Vaccine, Encephalitis, Reactivation, Neurological sequelae

## Abstract

The varicella zoster virus (VZV) is a latent viral infection and its reactivation has been reported following different conditions such as immunosuppression. This study presents a confirmed case of VZV encephalitis following the first dose administration of the Sinopharm COVID-19 vaccine. A 63-year-old immunocompetent woman who developed VZV encephalitis after first dose administration of Sinopharm COVID-19 vaccine. A final diagnosis of VZV encephalitis was made based on positive CSF PCR results for VZV infection. Treatment was administered with acyclovir and she returned to normal life without any neurological sequelae. In this report, VZV reactivation and VZV encephalitis have been observed after COVID-19 vaccination; however, the results of this report should be considered with some caution, and continued post-vaccine surveillance of adverse events is recommended to explore whether any causal association with VZV reactivation is biologically plausible in this context, or if it is just a coincidence.

## Abbreviations

COVID-19: coronavirus disease 2019
RNA: ribonucleic acid
SARS-CoV-2: severe acute respiratory syndrome coronavirus-2
WHO: World Health Organization
ARDS: acute respiratory distress syndrome
MODS: multiple organ dysfunction syndromes
RT-PCR: real-time polymerase chain reaction
IgM: immunoglobulin M
IgG: immunoglobulin G
ESR: erythrocyte sedimentation rate
CRP: C-reactive protein
IL: interleukin
TNF: tumor necrosis factor
PNR: platelet-to-neuophil ratio
PLR: platelet-to-lymphocyte ratio
PMR: platelet-to-monocyte ratio
NLR: neutrophil-to-lymphocyte ratio
dNLR: derived NLR
NMR: neutrophil-to-monocyte ratio
MLR: monocyte-to-lymphocyte ratio
ELR: eosinophil-to-lymphocyte ratio
CLR: CRP-to-lymphocyte ratio
CT: computerized tomography
CBC: complete blood cell
WBC: white blood cell
RBC: red blood cell
Hb: hemoglobin
Hct: hematocrit
MCV: mean corpuscular volume
MCH: mean corpuscular Hb
MCHC: mean corpuscular Hb concentration
PCV: packed cell volume
RDW: red cell distribution width
Neut: neutrophil
Lymph: lymphocyte
Mono: monocyte
Eosin: eosinophil
PLT: platelet
P-LCR: platelet-large cell ratio
MPV: mean platelet volume
PDW: platelet distribution width
ELISA: enzyme-linked immunosorbent assay
RBD: receptor binding domain
ACE2: angiotensin-converting enzyme 2
NAI: naturally acquired immunity

## 1 Introduction

Reactivation of latent viral infections has been reported after immunosuppression, including Coronavirus Disease 2019 (COVID-19) patients who received immunomodulatory therapies [1,2]. The development of various vaccine platforms led to a decrease in mortality and morbidity of COVID-19 [3]. However, COVID-19 vaccinations have been associated with some adverse effects, such as reactivation of latent infections [4,5]. Reactivation of the varicella zoster virus (VZV) was reported in some cases following COVID-19 vaccination [4,6]. Here, we report a unique case of VZV encephalitis after COVID-19 vaccine administration.

### 1.1 Case presentation

A 63-year-old woman came to the emergency department with generalized headache, fatigue, and myalgia. The record of the history revealed that the patient received the first dose of Sinopharm/BBIBPCorV COVID-19 vaccine four days before admission. She had a history of hypertension and anemia (but did not take specific medications). As such, she had a history of chickenpox in childhood, but no history of vaccination for the herpes zoster virus. No prior history of herpes simplex virus (HSV) and COVID-19 infection was reported. On admission, she was conscious and her vital signs were as follows: body temperature: 37 °C, pulse rate: 98 bpm, respiration rate: 22 breaths/min, blood pressure: 110/78 mmHg and arterial oxygen saturation (O2 SAT) was 90 %. On physical examination, there were no abnormal focal neurological deficits. During hospitalization, she had periods of headache, shoulder pain, anorexia, nausea & vomiting, abdominal pain, constipation, back pain, leg pain, and altered mental status (she initially had confusion and later experienced occasional disorientation and delirium).

**Fig. 1:**
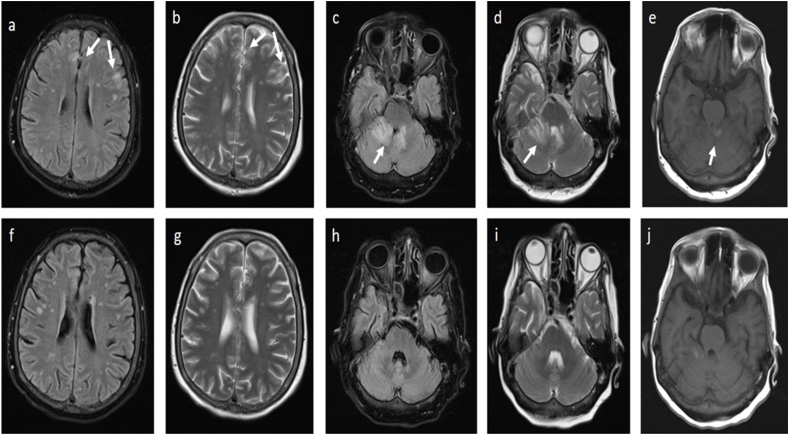
Post-COVID-19 vaccination magnetic resonance imaging (MRI) suggestive of encephalitis: Flair and T2 hypersignal changes in the cortical and subcortical areas of the frontoparietal lobes (a, b), cerebellar vermis, right cerebellar hemisphere and right middle cerebellar peduncle (c, d) without significant enhancement or restriction. Small focus of T1 hypersignality in the left side of the cerebellar hemisphere suggestive of hemorrhage (e). Post-treatment brain MRI (at 28 days after admission) showed subtle changes in MRI, in which signal changes disappeared (f, g, h, i, j).

The diagnosis was made by spiral high resolution computed tomography (HRCT) scans of the lung to investigate the possibility of COVID-19 infection. Significant findings on the patient's HRCT scan are increased haziness at the base of both lower lobes, a 5 mm nodule at the base of the right lower lobe, and a prevertebral soft tissue lesion on the right side in favor of extra medullary hematopoiesis. The COVID-19 real-time polymerase chain reaction (PCR) test was negative. Additionally, the results of the primary blood tests showed pancytopenia (WBC: 1800/microliter (Mic), RBC: 344000/Mi, Hb: 10.5 g/dl). Four days after admission, she developed multiple painful vesicular lesions and erythematous patches on the right posterior chest. The patient was examined by a dermatologist and the diagnosis of herpes zoster was confirmed. Due to the worsening of symptoms, especially persistent headache, nausea, and vomiting, brain computed tomography (CT) and neurology consultation were performed. Brain CT showed hypodensity in the cerebellum. Neurological consultation recommended brain magnetic resonance imaging (MRI). Hence, a brain MRI with and without gadolinium was performed. Extensive signal changes in the cortex and cerebellum were reported in MRI, as well as necrotizing lesions, suggesting the diagnosis of encephalitis (Fig. 1, a j). Consequently, the patient received broad-spectrum antibiotics (ceftriaxone and vancomycin), antiviral (acyclovir), corticosteroids (dexamethasone) and a lumbar puncture (LP) procedure. The collected cerebrospinal fluid (CSF) sample was sent to the laboratory for PCR of HSV-1, HSV-2, CMV, VZV, and severe acute respiratory syndrome coronavirus-2 (SARS-COV-2). Subsequently, she was transferred to the ICU (Peymaniyeh hospital, Jahrom, Iran) for further evaluation with the preliminary diagnosis of encephalitis. In the ICU, the patient's delirium worsened and the patient's consciousness occasionally decreased. The CSF analysis demonstrated pleocytosis, a WBC count of 865 per mm^3^ with neutrophils predominance (neutrophils 62%, lymphocytes 38%), a slightly elevated protein level (60.6 mg/dl, normal range 15–45) and glucose levels (32 mg/dl, normal range 40–75). On day 17 after admission, the CSF PCR result for VZV DNA was positive and the diagnosis of VZV encephalitis was confirmed (Table 1). After 33 days of hospitalization and receiving 28 days of acyclovir (Supplementary Table 1), the patient was discharged from the hospital in good general condition.

**Table 1:** Additional laboratory tests which were performed for the patient.

Test	Results	Normal range
Blood culture	Negative	–
Urine culture	Negative	–
FOB	Negative	–
HIV ELFA	Negative	–
HBSAg ELFA	Negative	–
HCV ELFA	Negative	–
CA 19-9	3.42 U/ml	Up to 37
CA 125(Immulite) (U/ml)	3.6 U/ml	0–14
anti-dsDNA IgM antibody	6.9	
anti-dsDNA IgG antibody	30.2	
anti-TPO antibodies	2.3 IU/ml	Up to 31.5

**FOB**: Fecal occult blood.

## 2 Discussion

VZV is a common neurotropic human-limited alpha-herpesvirus [7]. VZV causes varicella or chickenpox, usually in unvaccinated children [7], which presents as a generalized vesicular skin rash [7]. However, the virus remains latent in the sensory ganglia (dorsal root ganglia and cranial nerve ganglia, such as the trigeminal ganglia and the autonomic ganglia in the enteric nervous system (ENS)) [7]. Reactivation of the latent infection has been occurred spontaneously or following various conditions (eg, aging, immune suppression, infections, X-ray radiation, trauma, and malignancy) [7], resulted in localized skin lesions, called herpes zoster or shingles [7]. VZV is the second most common viral agent after HSV in causing encephalitis [8]. Different central nervous system (CNS) disorders, including meningitis, encephalitis, cerebellitis, arteritis, myelitis, vasculitis, and stroke-related syndromes have been reported following herpes zoster, most of which were reported from immunocompromised individuals [9,10]. The most common symptoms after VZV encephalitis were headache, fever, vomiting, altered levels of consciousness, and seizures [9], which were observed in the present case. The diagnosis of VZV encephalitis is based on CSF analysis, PCR, brain CT, and MRI findings [9]. Acyclovir is the choice drug for treatment of VZV [9]. CSF pleocytosis is common in viral encephalitis, which refers to an increase in the number of white blood cells (WBC) (>5 x 10^9/L) [11]. CSF pleocytosis was also observed in the present case.

Various reports of VZV infection [[12], [13], [14]] or VZV encephalitis [[15], [16], [17]] have been reported after COVID-19 infection [2]. As such, cases of HSV encephalitis [18] and VZV meningitis [19] have also been reported after COVID-19 vaccination. The downregulation of natural killer (NK cells) group 2D (NKG2D) ligands (which help prevent autoreactivity of NK cells against host tissues) occurs during HSV and VZV infections, which can contribute to viral latency and evasion from NK cells reactions [18]. However, disruption of this balance can occur during stress-inducing conditions, such as hypoxia and viral infections such as SARS-COV-2 infection, which consequently lead to viral reactivation [18]. It has been suggested that COVID-19 vaccination can initiate cytokine release and immune response cascades, which potentially disrupt the function of CD4^+^ and CD8^+^ T cells that triggers VZV reactivation [19]. Furthermore, certain cytokines (such as IL-1, IL-6, TNF-α, and prostaglandins) can be released into the bloodstream, potentially involved in the induction of encephalitis [20]. Post vaccination encephalitis is associated with adjuvant-induced autoimmune/inflammatory syndrome induced by adjuvants (ASIA) [15]. ASIA can present with a wide range of clinical symptoms, including CNS involvements [15].

Although VZV encephalitis has been reported after COVID-19 infection [21], to the best of our knowledge, this is the first report of VZV encephalitis after COVID-19 vaccination.

As VZV encephalitis after COVID-19 vaccination is a very rare and perhaps random event, a causal relationship between these two events cannot be proved based on a single case. It is important to monitor vaccinated people for potential side effects of COVID-19 vaccination. However, healthcare providers should be aware of the initial symptoms of VZV encephalitis after vaccination and take timely interventions to prevent adverse outcomes.

## Ethical approval

Informed consent was obtained from the patient for the PUBLICATION of all of their data and/or images. The Ethics Committee of Jahrom University of Medical Sciences approved the case for publication (Ethic code: IR.JUMS.REC.1402.106).

## Data availability statement

All data were included in article/supplement.

## Funding

This research did not receive any specific grants from funding agencies in the public, commercial or nonprofit sectors.

## CRediT authorship contribution statement

**Sanaz Rezaeian:** Writing – original draft, Methodology, Investigation, Data curation. **Fatemeh Rahmanian:** Validation, Methodology, Investigation, Data curation. **Zohre Rajabpour:** Validation, Methodology, Investigation. **Ali Taghipour:** Validation, Supervision, Methodology, Investigation. **Mirza Ali Mofazzal Jahromi:** Validation, Methodology, Investigation. **Abdolvahab Rahmanian:** Validation, Methodology, Investigation. **Heshmatollah Shakeri:** Validation, Methodology, Investigation. **Navid Kalani:** Validation, Methodology, Investigation. **Maryam Jalali Jahromi:** Validation, Supervision, Resources, Project administration, Methodology, Investigation, Formal analysis, Data curation, Conceptualization. **Amir Abdoli:** Writing – review & editing, Visualization, Validation, Supervision, Investigation, Formal analysis, Data curation, Conceptualization.

## Declaration of competing interest

The authors declare the following financial interests/personal relationships which may be considered as potential competing interests. Amir Abdoli reports a relationship with Jahrom University of Medical Science that includes: non-financial support. The authors have no competing interests to declare. Other authors have no known competing financial interests or personal relationships that could influence the work reported in this paper.
