# Supplementary material for: Case report: Varicella zoster virus encephalitis following COVID-19 vaccination in an immunocompetent individual
Source: Heliyon. 2024 Mar 30;10(7):e28703. doi: 10.1016/j.heliyon.2024.e28703 (PMC11002061; doi:10.1016/j.heliyon.2024.e28703)
Supplement: Multimedia component 1 [file mmc1.docx]

**Supplementary Table 1.** A summary of prescription drugs information for the patient.

| **Drugs** | **Drug Form** | **Drug**  **Name** | **Drug Dosage** | **Route for ingestion** | **Frequency** | **Duration** | **timing of commencement** |
| --- | --- | --- | --- | --- | --- | --- | --- |
| **antibiotics** | Amp | Ceftriaxone | 2 gr | IV | Q12h | For 14 days | On day 5 after admission |
|  | Amp | Vancomycin | 1 gr | IV | Q12h | For 14 days | On day 5 after admission |
| **antiviral** | Amp | Acyclovir | 600 mg | IV | Q8h | For 21 days | On day 5 after admission |
| **corticosteroids** | Amp | Dexamethasone | 9 mg | IV | Q6h | For 4 days | On day 5 after admission |
